# 1,1,3,3-Tetra-*tert*-butyl-2,2-diisopropyl-4,4-diphenyl­cyclo­tetra­silane

**DOI:** 10.1107/S160053681205074X

**Published:** 2012-12-22

**Authors:** Takayoshi Kuribara, Soichiro Kyushin

**Affiliations:** a Department of Chemistry and Chemical Biology, Graduate School of Engineering, Gunma University, Kiryu, Gunma 376-8515, Japan

## Abstract

The molecule in the structure of the title compound, C_34_H_60_Si_4_, lies on a twofold rotation axis that passes through the two Si atoms, resulting in a planar cyclo­tetra­silane ring. The dihedral angle between the cyclo­tetra­silane ring and the phenyl ring is 68.20 (5)°. The Si—Si bonds [2.4404 (8) and 2.4576 (8) Å] are longer than a standard Si—Si bond (2.34 Å) and the C—Si—C bond angle [97.07 (14)°] of the phenyl-substituted Si atom is smaller than the tetra­hedral bond angle (109.5°). These long bonds and small bond angle are favorable for reducing the steric hindrance among the bulky substituents.

## Related literature

For background to and applications of phenyl-substituted oligosilanes, see: Hinch & Krc (1957 ▶); Matsumoto & Tanaka (2008 ▶). For a related structure of a cyclo­tetra­silane without phenyl groups, see: Kyushin *et al.* (1995 ▶).
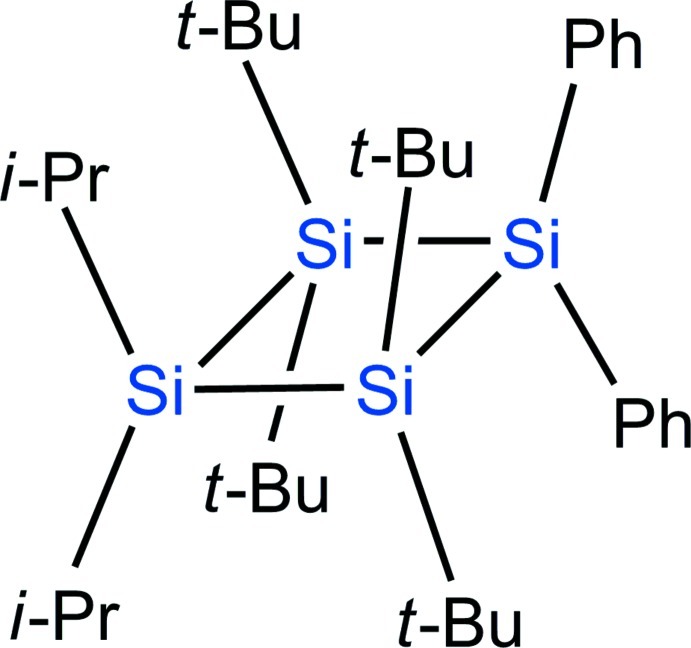


## Experimental

### 

#### Crystal data

C_34_H_60_Si_4_
*M* *_r_* = 581.18
Monoclinic, 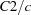

*a* = 11.9477 (9) Å
*b* = 17.6585 (12) Å
*c* = 17.0422 (13) Å
β = 104.9394 (8)°
*V* = 3474.0 (4) Å^3^
*Z* = 4
Mo *K*α radiation
μ = 0.19 mm^−1^
*T* = 173 K
0.50 × 0.40 × 0.20 mm

#### Data collection

Rigaku R-AXIS IV imaging plate diffractometer
Absorption correction: multi-scan (*REQAB*; Jacobson, 1998 ▶) *T*_min_ = 0.910, *T*_max_ = 0.963
8534 measured reflections
2917 independent reflections
2899 reflections with *I* > 2σ(*I*)
*R*_int_ = 0.025

#### Refinement

*R*[*F* ^2^ > 2σ(*F* ^2^)] = 0.048
*wR*(*F* ^2^) = 0.095
*S* = 1.24
2917 reflections
181 parameters
H-atom parameters constrained
Δρ_max_ = 0.24 e Å^−3^
Δρ_min_ = −0.20 e Å^−3^

### 

Data collection: *CrystalClear* (Rigaku, 2003 ▶); cell refinement: *CrystalClear*; data reduction: *CrystalClear*; program(s) used to solve structure: *SIR2004* (Burla *et al.*, 2005 ▶); program(s) used to refine structure: *SHELXL97* (Sheldrick, 2008 ▶); molecular graphics: *ORTEP-3* (Farrugia, 2012 ▶); software used to prepare material for publication: *SHELXL97* and *Yadokari-XG 2009* (Kabuto *et al.*, 2009 ▶).

## Supplementary Material

Crystal structure: contains datablock(s) global, I. DOI: 10.1107/S160053681205074X/is5224sup1.cif

Structure factors: contains datablock(s) I. DOI: 10.1107/S160053681205074X/is5224Isup2.hkl

Supplementary material file. DOI: 10.1107/S160053681205074X/is5224Isup3.cml

Additional supplementary materials:  crystallographic information; 3D view; checkCIF report

## Figures and Tables

**Fig. 1.**
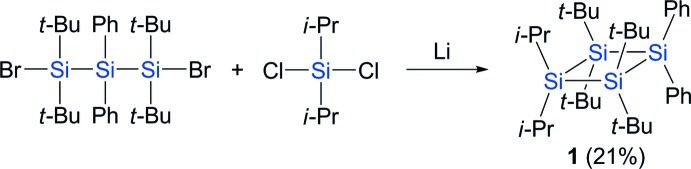
Synthesis of 1.

**Fig. 2.**
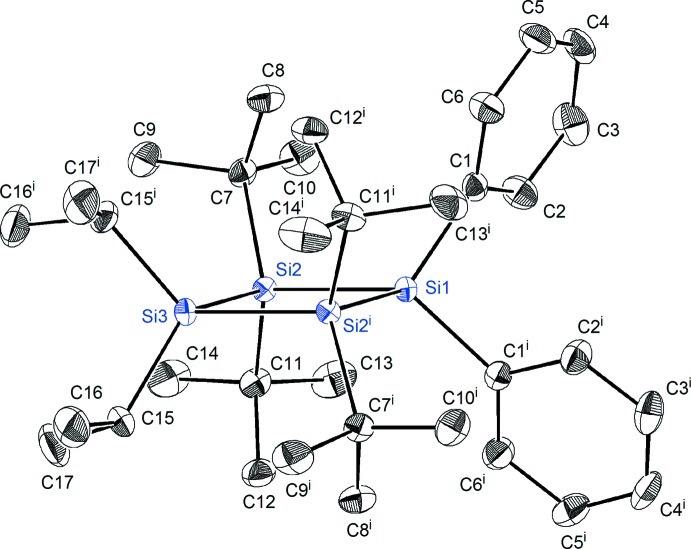
The molecular structure of 1, showing 50% probability displacement ellipsoids. [Symmetry code: (i) –x, y, –z + 3/2.]

**Fig. 3.**
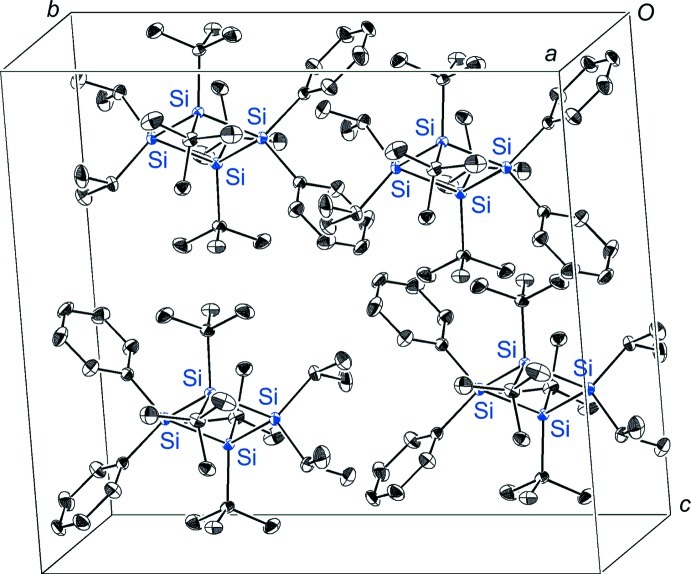
Packing diagram of 1, showing 50% probability displacement ellipsoids.

## supplementary crystallographic information

### Comment

Birefringent materials have a wide range of optical applications. Single crystals of calcium carbonate and barium borate have been well known as inorganic birefringent materials. Organic single crystals such as urea have also been known to show birefringence. Since birefringence is related to crystal structures, studies on molecular structures and packing in a crystal are important. In 1957, birefringence of tetrakis(4-phenylphenyl)silane was reported (Hinch & Krc, 1957). Recently, birefringence of single crystals of phenyl-substituted linear oligosilanes and their application to polarizers have been reported (Matsumoto & Tanaka, 2008). From these results, crystals of phenyl-substituted silicon compounds seem interesting as potential optical materials. We report herein the synthesis and X-ray crystal analysis of a phenyl-substituted cyclotetrasilane.

The coupling of 1,3-dibromo-1,1,3,3-tetra-*tert*-butyl-2,2-diphenyltrisilane and dichlorodiisopropylsilane with lithium in tetrahydrofuran (THF) gave 1,1,3,3-tetra-*tert*-butyl-2,2-diisopropyl-4,4-diphenylcyclotetrasilane (1) in 21% yield (Fig. 1). The molecular structure of 1 is shown in Fig. 2. Compound 1 has the crystallographic *C*2 symmetry, and therefore the cyclotetrasilane ring has a completely planar structure. The silicon–silicon bonds [2.4404 (8) and 2.4576 (8) Å] are longer than the standard silicon–silicon bond (2.34 Å). The C1—Si1—C1^i^ bond angle [97.07 (14)°] is smaller than the tetrahedral bond angle (109.5°), while the C7—Si2—C11 [111.39 (10)°] and C15—Si3—C15^i^ [106.97 (15)°] bond angles are within normal values. The long silicon–silicon bonds and the small carbon–silicon–carbon bond angle are favorable for reducing the steric hindrance among bulky substituents.

Packing diagram of 1 is shown in Fig. 3. Four molecules are present in a unit cell. All cyclotetrasilane rings are oriented toward the same direction with the line through the Si1 and Si3 atoms parallel to the *b* axis. There is no intermolecular π–π interaction among phenyl groups.

### Experimental

A mixture of 1,3-dibromo-1,1,3,3-tetra-*tert*-butyl-2,2-diphenyltrisilane (5.00 g, 7.98 mmol), dichlorodiisopropylsilane (2.24 g, 12.1 mmol) and lithium (0.28 g, 40 mmol) in THF (50 ml) was stirred at room temperature for 1 day. The solvent was removed under reduced pressure. The residue was dissolved in hexane and passed through a short column of silica gel. After the silica gel was washed with hexane, the eluent was changed to diethyl ether. The diethyl ether eluate was evaporated. Recrystallization of the residue from methanol–THF (*ca* 1:1) gave 1 (0.956 g, 21%) as colorless crystals. Single crystals were obtained from methanol–THF (*ca* 1:1) by slow evaporation.

M.p.: 210–211 °C. ^1^H NMR (400 MHz, CDCl_3_): *δ* 1.21 (s, 36H), 1.46 (d, 12H, *J* = 7.4 Hz), 1.94 (sept, 2H, *J* = 7.4 Hz), 7.14–7.18 (m, 6H), 7.68–7.70 (m, 4H). ^13^C NMR (76 MHz, CDCl_3_): *δ* 17.5, 24.7, 25.4, 32.8, 127.1, 127.4, 138.6, 142.1. ^29^Si NMR (119 MHz, CDCl_3_): *δ* -6.2, 14.0, 20.4. IR (KBr): 3050, 2950, 2920, 2850, 1470, 1420, 1390, 1360, 810, 730, 700 cm^-1^. MS (EI, 70 eV): *m*/*z* 580 (*M*^+^, 17), 360 (100).

### Refinement

All hydrogen atoms were generated at calculated positions and refined as riding atoms, with C—H = 0.95 (phenyl), 0.98 (methyl) or 1.00 (methine) Å, and with *U*_iso_(H) = 1.2*U*_eq_(phenyl C), 1.5*U*_eq_(methyl C) or 1.2*U*_eq_(methine C).

### Crystal data

**Table:**

C_34_H_60_Si_4_	*F*(000) = 1280
*M**_r_* = 581.18	*D*_x_ = 1.111 Mg m^−^^3^
Monoclinic, *C*2/*c*	Melting point = 483–484 K
Hall symbol: -C 2yc	Mo *K*α radiation, λ = 0.71073 Å
*a* = 11.9477 (9) Å	Cell parameters from 7676 reflections
*b* = 17.6585 (12) Å	θ = 1.2–28.3°
*c* = 17.0422 (13) Å	µ = 0.19 mm^−^^1^
β = 104.9394 (8)°	*T* = 173 K
*V* = 3474.0 (4) Å^3^	Prism, colourless
*Z* = 4	0.50 × 0.40 × 0.20 mm

### Data collection

**Table:**

Rigaku R-AXISIV imaging plate diffractometer	2917 independent reflections
Radiation source: rotating anode	2899 reflections with *I* > 2σ(*I*)
Graphite monochromator	*R*_int_ = 0.025
Detector resolution: 10.00 pixels mm^-1^	θ_max_ = 25.0°, θ_min_ = 2.1°
ω scans	*h* = −14→14
Absorption correction: multi-scan (*REQAB*; Jacobson, 1998)	*k* = −18→20
*T*_min_ = 0.910, *T*_max_ = 0.963	*l* = −20→20
8534 measured reflections	

### Refinement

**Table:**

Refinement on *F*^2^	Primary atom site location: structure-invariant direct methods
Least-squares matrix: full	Secondary atom site location: difference Fourier map
*R*[*F*^2^ > 2σ(*F*^2^)] = 0.048	Hydrogen site location: inferred from neighbouring sites
*wR*(*F*^2^) = 0.095	H-atom parameters constrained
*S* = 1.24	*w* = 1/[σ^2^(*F*_o_^2^) + (0.0127*P*)^2^ + 7.2943*P*] where *P* = (*F*_o_^2^ + 2*F*_c_^2^)/3
2917 reflections	(Δ/σ)_max_ = 0.001
181 parameters	Δρ_max_ = 0.24 e Å^−^^3^
0 restraints	Δρ_min_ = −0.20 e Å^−^^3^

### Special details

**Table:**

Geometry. All e.s.d.'s (except the e.s.d. in the dihedral angle between two l.s. planes) are estimated using the full covariance matrix. The cell e.s.d.'s are taken into account individually in the estimation of e.s.d.'s in distances, angles and torsion angles; correlations between e.s.d.'s in cell parameters are only used when they are defined by crystal symmetry. An approximate (isotropic) treatment of cell e.s.d.'s is used for estimating e.s.d.'s involving l.s. planes.
Refinement. Refinement of *F*^2^ against ALL reflections. The weighted *R*-factor *wR* and goodness of fit *S* are based on *F*^2^, conventional *R*-factors *R* are based on *F*, with *F* set to zero for negative *F*^2^. The threshold expression of *F*^2^ > 2σ(*F*^2^) is used only for calculating *R*-factors(gt) *etc.* and is not relevant to the choice of reflections for refinement. *R*-factors based on *F*^2^ are statistically about twice as large as those based on *F*, and *R*-factors based on ALL data will be even larger.

### Fractional atomic coordinates and isotropic or equivalent isotropic displacement parameters (Å^2^)

**Table:**

	*x*	*y*	*z*	*U*_iso_*/*U*_eq_	
Si1	0.0000	0.32355 (5)	0.7500	0.01350 (19)	
Si2	0.09403 (5)	0.22594 (3)	0.68813 (3)	0.01324 (15)	
Si3	0.0000	0.12695 (5)	0.7500	0.01475 (19)	
C1	−0.07934 (19)	0.39557 (13)	0.67052 (13)	0.0179 (5)	
C2	−0.0078 (2)	0.45046 (14)	0.64962 (15)	0.0269 (6)	
H1	0.0725	0.4498	0.6763	0.032*	
C3	−0.0487 (2)	0.50564 (15)	0.59179 (17)	0.0330 (6)	
H2	0.0031	0.5416	0.5791	0.040*	
C4	−0.1657 (2)	0.50818 (15)	0.55245 (15)	0.0307 (6)	
H3	−0.1947	0.5455	0.5122	0.037*	
C5	−0.2392 (2)	0.45599 (15)	0.57252 (16)	0.0313 (6)	
H4	−0.3196	0.4576	0.5462	0.038*	
C6	−0.1968 (2)	0.40088 (14)	0.63102 (15)	0.0262 (5)	
H5	−0.2494	0.3659	0.6444	0.031*	
C7	0.03683 (19)	0.22973 (13)	0.56985 (13)	0.0191 (5)	
C8	−0.0961 (2)	0.23931 (15)	0.54491 (14)	0.0259 (5)	
H6	−0.1243	0.2356	0.4857	0.039*	
H7	−0.1165	0.2890	0.5629	0.039*	
H8	−0.1318	0.1994	0.5703	0.039*	
C9	0.0639 (2)	0.15671 (16)	0.52878 (15)	0.0331 (6)	
H9	0.0373	0.1622	0.4697	0.050*	
H10	0.0240	0.1139	0.5462	0.050*	
H11	0.1476	0.1476	0.5444	0.050*	
C10	0.0887 (2)	0.29704 (15)	0.53389 (15)	0.0307 (6)	
H12	0.1716	0.2884	0.5404	0.046*	
H13	0.0779	0.3436	0.5623	0.046*	
H14	0.0497	0.3020	0.4760	0.046*	
C11	0.26218 (18)	0.23095 (14)	0.72206 (14)	0.0204 (5)	
C12	0.3017 (2)	0.21501 (14)	0.81400 (14)	0.0253 (5)	
H15	0.2866	0.1618	0.8242	0.038*	
H16	0.2587	0.2477	0.8425	0.038*	
H17	0.3848	0.2254	0.8338	0.038*	
C13	0.3052 (2)	0.31047 (16)	0.70706 (17)	0.0347 (6)	
H18	0.3880	0.3148	0.7340	0.052*	
H19	0.2621	0.3486	0.7291	0.052*	
H20	0.2929	0.3186	0.6486	0.052*	
C14	0.3208 (2)	0.17278 (18)	0.67839 (17)	0.0379 (7)	
H21	0.3064	0.1865	0.6209	0.057*	
H22	0.2889	0.1223	0.6830	0.057*	
H23	0.4044	0.1724	0.7034	0.057*	
C15	0.0992 (2)	0.06194 (13)	0.82929 (15)	0.0241 (5)	
H24	0.1364	0.0948	0.8766	0.029*	
C16	0.0306 (3)	0.00034 (16)	0.86183 (17)	0.0387 (7)	
H25	0.0068	−0.0395	0.8209	0.058*	
H26	−0.0382	0.0231	0.8734	0.058*	
H27	0.0798	−0.0215	0.9118	0.058*	
C17	0.1985 (2)	0.02186 (16)	0.80303 (19)	0.0387 (7)	
H28	0.2509	−0.0020	0.8504	0.058*	
H29	0.2414	0.0591	0.7796	0.058*	
H30	0.1662	−0.0170	0.7624	0.058*	

### Atomic displacement parameters (Å^2^)

**Table:**

	*U*^11^	*U*^22^	*U*^33^	*U*^12^	*U*^13^	*U*^23^
Si1	0.0139 (4)	0.0121 (4)	0.0151 (4)	0.000	0.0047 (3)	0.000
Si2	0.0127 (3)	0.0149 (3)	0.0133 (3)	−0.0005 (2)	0.0055 (2)	−0.0008 (2)
Si3	0.0165 (4)	0.0118 (4)	0.0169 (4)	0.000	0.0061 (4)	0.000
C1	0.0219 (11)	0.0167 (12)	0.0162 (11)	0.0022 (9)	0.0072 (10)	−0.0007 (9)
C2	0.0260 (12)	0.0273 (14)	0.0279 (14)	0.0013 (10)	0.0081 (11)	0.0074 (10)
C3	0.0415 (15)	0.0258 (14)	0.0367 (15)	−0.0008 (11)	0.0189 (13)	0.0106 (11)
C4	0.0465 (16)	0.0230 (14)	0.0230 (13)	0.0109 (11)	0.0101 (12)	0.0100 (10)
C5	0.0273 (13)	0.0336 (15)	0.0298 (14)	0.0095 (11)	0.0012 (12)	0.0062 (11)
C6	0.0227 (12)	0.0271 (14)	0.0276 (13)	0.0002 (10)	0.0044 (11)	0.0060 (10)
C7	0.0236 (12)	0.0209 (12)	0.0143 (11)	0.0012 (9)	0.0078 (10)	−0.0023 (9)
C8	0.0242 (12)	0.0346 (15)	0.0179 (12)	0.0005 (10)	0.0033 (10)	0.0002 (10)
C9	0.0410 (15)	0.0343 (16)	0.0232 (13)	0.0076 (12)	0.0070 (12)	−0.0095 (11)
C10	0.0375 (14)	0.0359 (16)	0.0219 (13)	−0.0041 (11)	0.0135 (12)	0.0068 (11)
C11	0.0144 (10)	0.0271 (13)	0.0210 (12)	−0.0006 (9)	0.0068 (10)	−0.0040 (9)
C12	0.0192 (11)	0.0299 (14)	0.0240 (13)	0.0013 (10)	0.0005 (10)	−0.0042 (10)
C13	0.0288 (13)	0.0431 (17)	0.0327 (15)	−0.0176 (12)	0.0086 (12)	0.0011 (12)
C14	0.0210 (13)	0.058 (2)	0.0355 (16)	0.0079 (12)	0.0092 (12)	−0.0157 (14)
C15	0.0289 (12)	0.0170 (12)	0.0243 (13)	0.0059 (10)	0.0029 (11)	0.0024 (9)
C16	0.0570 (18)	0.0254 (15)	0.0335 (15)	−0.0005 (13)	0.0113 (14)	0.0090 (12)
C17	0.0319 (14)	0.0246 (15)	0.058 (2)	0.0119 (11)	0.0084 (14)	0.0004 (13)

### Geometric parameters (Å, º)

**Table:**

Si1—C1	1.921 (2)	C9—H11	0.9800
Si1—Si2	2.4404 (8)	C10—H12	0.9800
Si2—C11	1.944 (2)	C10—H13	0.9800
Si2—C7	1.956 (2)	C10—H14	0.9800
Si2—Si3	2.4576 (8)	C11—C14	1.539 (3)
Si3—C15	1.929 (2)	C11—C13	1.539 (3)
C1—C6	1.394 (3)	C11—C12	1.541 (3)
C1—C2	1.398 (3)	C12—H15	0.9800
C2—C3	1.382 (3)	C12—H16	0.9800
C2—H1	0.9500	C12—H17	0.9800
C3—C4	1.386 (4)	C13—H18	0.9800
C3—H2	0.9500	C13—H19	0.9800
C4—C5	1.376 (4)	C13—H20	0.9800
C4—H3	0.9500	C14—H21	0.9800
C5—C6	1.392 (3)	C14—H22	0.9800
C5—H4	0.9500	C14—H23	0.9800
C6—H5	0.9500	C15—C17	1.543 (3)
C7—C10	1.539 (3)	C15—C16	1.548 (3)
C7—C9	1.541 (3)	C15—H24	1.0000
C7—C8	1.544 (3)	C16—H25	0.9800
C8—H6	0.9800	C16—H26	0.9800
C8—H7	0.9800	C16—H27	0.9800
C8—H8	0.9800	C17—H28	0.9800
C9—H9	0.9800	C17—H29	0.9800
C9—H10	0.9800	C17—H30	0.9800
			
C1^i^—Si1—C1	97.07 (14)	H10—C9—H11	109.5
C1^i^—Si1—Si2	125.03 (7)	C7—C10—H12	109.5
C1—Si1—Si2	111.19 (6)	C7—C10—H13	109.5
Si2^i^—Si1—Si2	90.13 (4)	H12—C10—H13	109.5
C11—Si2—C7	111.39 (10)	C7—C10—H14	109.5
C11—Si2—Si1	113.26 (7)	H12—C10—H14	109.5
C7—Si2—Si1	110.10 (7)	H13—C10—H14	109.5
C11—Si2—Si3	117.16 (8)	C14—C11—C13	108.4 (2)
C7—Si2—Si3	112.93 (7)	C14—C11—C12	108.2 (2)
Si1—Si2—Si3	90.27 (3)	C13—C11—C12	107.92 (19)
C15^i^—Si3—C15	106.97 (15)	C14—C11—Si2	112.95 (16)
C15^i^—Si3—Si2	112.89 (7)	C13—C11—Si2	110.84 (17)
C15—Si3—Si2	117.23 (7)	C12—C11—Si2	108.42 (14)
Si2^i^—Si3—Si2	89.33 (4)	C11—C12—H15	109.5
C6—C1—C2	115.8 (2)	C11—C12—H16	109.5
C6—C1—Si1	129.59 (18)	H15—C12—H16	109.5
C2—C1—Si1	114.57 (17)	C11—C12—H17	109.5
C3—C2—C1	122.9 (2)	H15—C12—H17	109.5
C3—C2—H1	118.6	H16—C12—H17	109.5
C1—C2—H1	118.6	C11—C13—H18	109.5
C2—C3—C4	119.7 (2)	C11—C13—H19	109.5
C2—C3—H2	120.2	H18—C13—H19	109.5
C4—C3—H2	120.2	C11—C13—H20	109.5
C5—C4—C3	119.1 (2)	H18—C13—H20	109.5
C5—C4—H3	120.4	H19—C13—H20	109.5
C3—C4—H3	120.4	C11—C14—H21	109.5
C4—C5—C6	120.6 (2)	C11—C14—H22	109.5
C4—C5—H4	119.7	H21—C14—H22	109.5
C6—C5—H4	119.7	C11—C14—H23	109.5
C5—C6—C1	121.9 (2)	H21—C14—H23	109.5
C5—C6—H5	119.1	H22—C14—H23	109.5
C1—C6—H5	119.1	C17—C15—C16	107.4 (2)
C10—C7—C9	108.18 (19)	C17—C15—Si3	116.68 (18)
C10—C7—C8	107.3 (2)	C16—C15—Si3	112.50 (17)
C9—C7—C8	106.7 (2)	C17—C15—H24	106.6
C10—C7—Si2	111.55 (16)	C16—C15—H24	106.6
C9—C7—Si2	112.41 (16)	Si3—C15—H24	106.6
C8—C7—Si2	110.42 (14)	C15—C16—H25	109.5
C7—C8—H6	109.5	C15—C16—H26	109.5
C7—C8—H7	109.5	H25—C16—H26	109.5
H6—C8—H7	109.5	C15—C16—H27	109.5
C7—C8—H8	109.5	H25—C16—H27	109.5
H6—C8—H8	109.5	H26—C16—H27	109.5
H7—C8—H8	109.5	C15—C17—H28	109.5
C7—C9—H9	109.5	C15—C17—H29	109.5
C7—C9—H10	109.5	H28—C17—H29	109.5
H9—C9—H10	109.5	C15—C17—H30	109.5
C7—C9—H11	109.5	H28—C17—H30	109.5
H9—C9—H11	109.5	H29—C17—H30	109.5
			
C1^i^—Si1—Si2—C11	−3.63 (12)	C3—C4—C5—C6	−0.6 (4)
C1—Si1—Si2—C11	112.02 (11)	C4—C5—C6—C1	−0.9 (4)
Si2^i^—Si1—Si2—C11	−119.92 (8)	C2—C1—C6—C5	2.0 (4)
C1^i^—Si1—Si2—C7	−129.08 (11)	Si1—C1—C6—C5	−177.9 (2)
C1—Si1—Si2—C7	−13.43 (11)	C11—Si2—C7—C10	−51.24 (19)
Si2^i^—Si1—Si2—C7	114.63 (8)	Si1—Si2—C7—C10	75.27 (16)
C1^i^—Si1—Si2—Si3	116.29 (8)	Si3—Si2—C7—C10	174.50 (14)
C1—Si1—Si2—Si3	−128.06 (7)	C11—Si2—C7—C9	70.49 (19)
Si2^i^—Si1—Si2—Si3	0.0	Si1—Si2—C7—C9	−163.01 (15)
C11—Si2—Si3—C15^i^	−124.05 (11)	Si3—Si2—C7—C9	−63.78 (18)
C7—Si2—Si3—C15^i^	7.41 (11)	C11—Si2—C7—C8	−170.44 (16)
Si1—Si2—Si3—C15^i^	119.45 (8)	Si1—Si2—C7—C8	−43.93 (18)
C11—Si2—Si3—C15	0.95 (12)	Si3—Si2—C7—C8	55.30 (18)
C7—Si2—Si3—C15	132.41 (11)	C7—Si2—C11—C14	−51.3 (2)
Si1—Si2—Si3—C15	−115.55 (9)	Si1—Si2—C11—C14	−176.05 (16)
C11—Si2—Si3—Si2^i^	116.50 (8)	Si3—Si2—C11—C14	80.87 (19)
C7—Si2—Si3—Si2^i^	−112.04 (8)	C7—Si2—C11—C13	70.59 (18)
Si1—Si2—Si3—Si2^i^	0.0	Si1—Si2—C11—C13	−54.17 (17)
C1^i^—Si1—C1—C6	−127.9 (2)	Si3—Si2—C11—C13	−157.25 (14)
Si2^i^—Si1—C1—C6	−5.8 (3)	C7—Si2—C11—C12	−171.14 (15)
Si2—Si1—C1—C6	100.2 (2)	Si1—Si2—C11—C12	64.11 (17)
C1^i^—Si1—C1—C2	52.17 (16)	Si3—Si2—C11—C12	−38.97 (18)
Si2^i^—Si1—C1—C2	174.29 (14)	C15^i^—Si3—C15—C17	74.54 (19)
Si2—Si1—C1—C2	−79.77 (18)	Si2^i^—Si3—C15—C17	−155.06 (17)
C6—C1—C2—C3	−1.9 (4)	Si2—Si3—C15—C17	−53.4 (2)
Si1—C1—C2—C3	178.1 (2)	C15^i^—Si3—C15—C16	−50.24 (16)
C1—C2—C3—C4	0.5 (4)	Si2^i^—Si3—C15—C16	80.16 (18)
C2—C3—C4—C5	0.7 (4)	Si2—Si3—C15—C16	−178.14 (15)

Symmetry code: (i) −*x*, *y*, −*z*+3/2.
